# Genetic Factors in the Pathogenesis of Nonalcoholic Fatty Liver and Steatohepatitis

**DOI:** 10.1155/2015/460190

**Published:** 2015-07-27

**Authors:** Paola Dongiovanni, Stefano Romeo, Luca Valenti

**Affiliations:** ^1^Internal Medicine and Metabolic Diseases, Fondazione IRCCS Ca' Granda Ospedale Maggiore Policlinico, Via Francesco Sforza 35, 20122 Milan, Italy; ^2^Wallenberg Laboratory, Department of Molecular and Clinical Medicine, Sahlgrenska Academy at the University of Gothenburg, Medicinaregatan 3, 40530 Gothenburg, Sweden; ^3^Clinical Nutrition Unit, Department of Medical and Surgical Sciences, University Magna Graecia, Viale Europa, 88100 Catanzaro, Italy; ^4^Department of Pathophysiology and Transplantation, University of Milan, Via Francesco Sforza 35, 20122 Milan, Italy

## Abstract

Liver fat accumulation generally related to systemic insulin resistance characterizes nonalcoholic fatty liver disease (NAFLD), which in the presence of nonalcoholic steatohepatitis (NASH) can progress towards cirrhosis and hepatocellular carcinoma. Due to the epidemic of obesity, NAFLD is now the most frequent liver disease in Western countries. Epidemiological, familial, and twin studies provide evidence for a strong genetic component of NAFLD susceptibility. Recently, genome-wide association studies led to the identification of the major inherited determinants of hepatic fat accumulation: *patatin-like phospholipase domain-containing 3 (PNPLA3)* I148M gene and *transmembrane 6 superfamily member 2 (TM6SF2)* E167K gene variants, involved in lipid droplets remodelling and very low-density lipoproteins secretion, are the major determinants of interindividual differences in liver steatosis, and susceptibility to progressive NASH. In this review, we aimed to provide an overview of recent insights into the genetics of hepatic fat accumulation and steatohepatitis.

## 1. Introduction

Nonalcoholic fatty liver disease (NAFLD) is defined by liver fat deposition in the absence of excessive alcohol intake. NAFLD is frequently present in obese individuals and it is also related to metabolic changes such as systemic insulin resistance [1]. In susceptible individuals, hepatic steatosis may result in oxidative hepatocellular damage, inflammation, and activation of fibrogenesis, namely, nonalcoholic steatohepatitis (NASH) [2]. This condition potentially progresses towards liver cirrhosis and hepatocellular carcinoma [3]. Due to the epidemic of obesity and the advances in the prevention and treatment of viral hepatitis NAFLD is now the most frequent liver disease and the leading cause of altered liver enzymes in Western countries [4, 5]. Subsequently, NASH will become the leading cause of end-stage liver disease, liver transplantation, and hepatocellular carcinoma within the next decades.

Epidemiological, familial, and twin studies provide evidence for heritability of hepatic fat content, NAFLD, and* bona fide* metabolic cirrhosis [6, 7]. In the last years, the genetic determinants of steatosis are being unrevealed using genome-wide association studies. These studies have identified* patatin-like phospholipase domain-containing 3 (PNPLA3)* gene variant, involved in hepatocellular lipid droplets remodelling and very low-density lipoprotein (VLDL) secretion, as a major determinant of interindividual and ethnicity-related differences in hepatic fat content [8, 9]. The* transmembrane 6 superfamily member 2 (TM6SF2)* E167K gene variant, interfering with VLDL secretion, which may be stimulated in early NAFLD [10], has recently been shown to increase susceptibility to progressive NASH by compartmentalization of lipids within hepatocytes [11, 12]. Furthermore, case-control studies demonstrated a role of other genetic variants implicated in inflammation [13], insulin signaling [14], oxidative stress, iron metabolism [15–18], and fibrogenesis [19] in the progression of fatty liver towards NASH associated fibrosis.

Newly identified genetic risk variants could provide a useful tool for the clinical management and prognosis of patients with NAFLD. They may also lead to the identification of drugs for treating NASH, a condition for which specific pharmacological treatment is still lacking.

The main purpose of this narrative review is to provide an overview of recent insights into the genetics of hepatic fat accumulation and steatohepatitis, specifically focusing on inherited variants regulating lipid metabolism. These recent advances have contributed in a short time to unravel the pathogenesis of NAFLD and may soon translate into therapeutic advances. For a comprehensive overview of all studies related to NAFLD and NASH genetics see the recent review by our group [20].

## 2. Pathophysiology of NAFLD and NASH: The Current View

**Figure 1:**
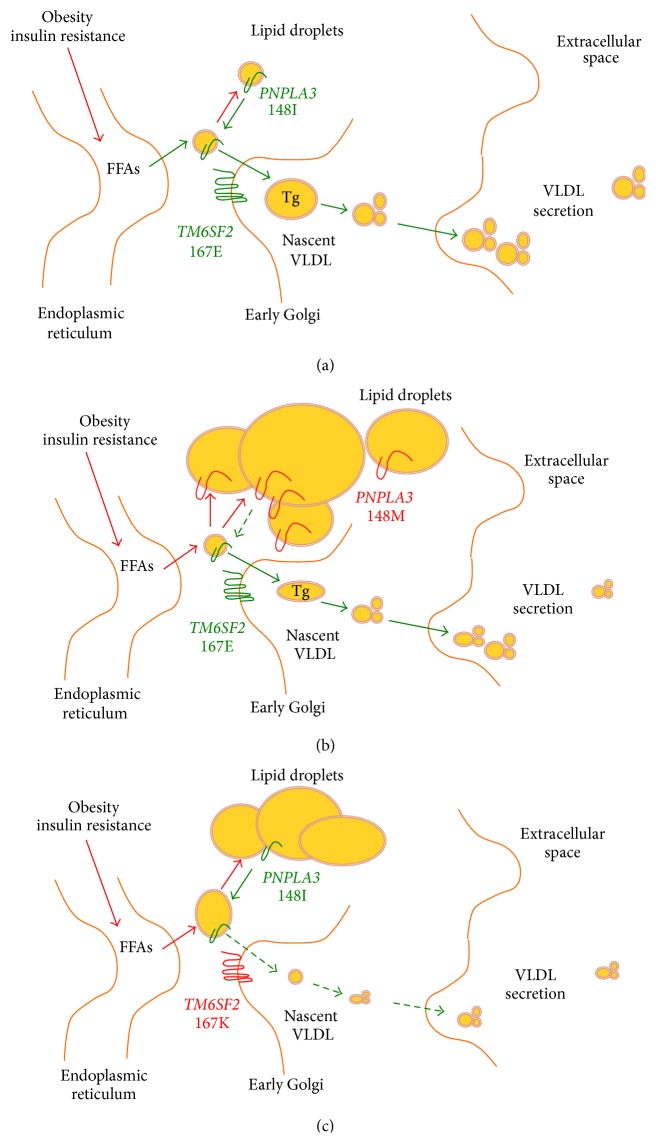
Molecular genetics of NASH. (a) NAFLD is characterized by the hepatic fat accumulation in lipid droplets resulting from an unbalance between triglycerides acquisition and secretion. FFA stored as triglycerides during hepatic steatosis derive from peripheral lipolysis related to adipose tissue insulin resistance, followed by* de novo* lipogenesis induced by hyperinsulinemia, and excessive food intake. In the liver, FFA can be catabolized through *β*-oxidation and reesterification to triglycerides and stored as lipid droplets or exported as VLDL. (b)* PNPLA3* I148M variant is attached on the surface of lipid droplets reducing triglyceride breakdown leading to lipid retention in the hepatocyte lipid droplet. (c)* TM6SF2* E167K variant reduces triglycerides secretion through VLDL, leading to hepatocellular retention of lipids.

The acronym NAFLD defines a wide spectrum of liver disease characterized by hepatic fat accumulation in the form of triglycerides exceeding 5% of liver mass in the absence of significant alcohol consumption. NAFLD may remain uncomplicated or progress into severe hepatitis characterized by severe steatosis, lobular inflammation, and hepatocellular damage and apoptosis with activation of fibrogenesis [21]. The final outcome of NAFLD is life threatening conditions, namely, cirrhosis and hepatocellular carcinoma [3]. Hepatic fat accumulation results from an unbalance between triglycerides acquisition, synthesis, utilization, and secretion [22, 23] and it represents the safest way to store fatty acids (FFAs) in the liver [24]. Several lines of evidence support a model where the excess in hepatocellular triglycerides derives from increased peripheral lipolysis [25] due to adipose tissue insulin resistance [26], an increased hepatic lipogenesis due to hyperinsulinemia, and an excessive food intake (Figure 1(a)). Indeed, the major determinant of NAFLD is systemic insulin resistance [1, 27]. Steatosis* per se* may then in turn worsen hepatic insulin resistance contributing to metabolic disturbances and cardiovascular damage [28, 29]. Reduction in hepatocellular triglycerides secretion through VLDL [23] and in utilization due to mitochondrial damage is also involved in hepatic fat accumulation.

The development of NASH has been classically ascribed to the occurrence of multiple parallel “second-hits,” leading to the activation of inflammation, in the context of hepatic steatosis [30, 31]. This second insult may be related to a variety of conditions: (a) direct hepatic lipotoxicity, (b) hepatocellular oxidative stress secondary to free radicals produced during *β*- and omega-oxidation of FFAs, (c) inflammation triggered by endotoxin engaging Toll-like receptor-4 in Kupffer cells and hepatocytes due to increased intestinal permeability, (d) qualitative and quantitative changes in gut microbiota [32–34], (e) release of cytokines by the hepatic stellate cells, and (f) endoplasmic reticulum stress. All these conditions lead in the end to inflammation, cellular damage, and activation of fibrogenesis in the liver [35].

## 3. Heritability of NAFLD and NASH

Epidemiological, familial, and twin studies [6, 7] and clinical case series showing familial clustering support a strong heritability component in NAFLD and NASH [36]. Twin studies show that ALT levels, mostly reflecting liver fat content in the absence of alcohol abuse or viral hepatitis, are a heritable trait explained up to 60% by genetic factors [37]. A study population among Danish twins identified substantial heritability (35–61%) for levels of aminotransferases [38].

Ethnic differences have been reported in the prevalence of NAFLD and NASH [4, 39]. Hispanics are at higher risk than individuals of European descent, whereas those from African descent are protected from these conditions irrespective of diabetes and excess body weight [7, 40].

## 4. *PNPLA3* I148M Mutation Is the Major Genetic Determinant of NAFLD and NASH

The major determinant of the interindividual and ethnicity-related differences in hepatic fat content was identified by an exome wide association study. This is the rs738409 C>G single nucleotide polymorphism (SNP) in the* PNPLA*3 gene, encoding for the isoleucine to methionine substitution at position 148 (I148M) [8]. In humans* PNPLA3*, also called* adiponutrin*, encodes a 481 amino acid membrane protein localized in the endoplasmic reticulum and at the surface of lipid droplets. In human this protein has the highest expression in hepatic stellate cells, retina, and hepatocytes. In mice Pnpla3 is upregulated in the liver after feeding and during insulin resistance by fatty acids and the master regulator of lipogenesis SREBP-1c [41]. Although the mechanism underlying the progression to liver disease remains an area of active research, PNPLA3 has a triglyceride and retinyl-palmitate esterase activity [42–44]. The isoleucine to methionine substitution leads to a loss of function of these activities, of the enzyme, leading to changes in an impairment of lipid catabolism, lipid droplets remodelling, and VLDL secretions [42, 45, 46]. This would favour hepatocellular accumulation of triglycerides during insulin resistance (Figure 1(b)).

A robust association of the I148M variant with hepatic fat content has been confirmed in several studies both in adults [8, 47–58] and in developmental age [59–62]. Most importantly, in carriers of the I148M mutant protein environmental stressors, namely, obesity [63], abdominal fat [64, 65], excessive alcohol consumption [66], chronic viral hepatitis [67, 68], or iron overload [69], trigger progressive liver damage [58]. Dietary habits are also relevant; indeed, the magnitude of the increase in liver enzymes in I148M carriers is correlated to high dietary carbohydrate and sugar consumption [70–72] and increased omega6/omega3 polyunsaturated fatty acids ratio [73, 74]. Interestingly, the carriers of the* PNPLA3* I148M variant have a substantial increased risk in cirrhosis and hepatocellular carcinoma [66, 67, 75–84] independently of the predisposition to steatosis. This suggests that PNPLA3 contributes directly to the fibrogenesis and carcinogenesis [9, 57, 61, 83, 85]. Indeed, PNPLA3 retinyl-palmitate activity in hepatic stellate cells may influence hepatic regeneration and differentiation by altering availability of retinol, potent regulators of these phenomena [44].

To summarize the* PNPLA3* I148M variant is a robust genetic determinant of hepatic steatosis triggered by a number of environmental factors [9, 85] and the PNPLA3 associated steatohepatitis (PASH) may be mediated by a direct effect on hepatocyte and on hepatic stellate cells [86]. The mechanisms linking the I148M* PNPLA3* variant with liver disease progression and hepatocellular carcinoma development have recently been reviewed by our group [9, 83].

## 5. *TM6SF2* E167K and Very Low-Density Lipoproteins Secretion

In 2014, two exome and genome wide association studies were reported identifying the rs58542926 C > T genetic variant of the* transmembrane 6 superfamily member 2* gene (*TM6SF2*), which encodes the loss-of-function lysine (E) to glutamic acid (K) at position 167 substitution (E167K), as a determinant of hepatic triglyceride content, serum aminotransferases, and lower serum lipoproteins [11, 87]. The same studies demonstrated that silencing of* TM6SF2 *reduces secretion of VLDL resulting in intrahepatic retention of triglycerides and steatosis in mice and in hepatocytes* in vitro* [11, 87, 88].

Very recently, in large collaborative European study evaluating a large cross-sectional cohort of 1,201 individuals at risk of NASH it was demonstrated that the E167K variant is associated with the full spectrum of liver damage associated with hepatic fat accumulation, including NASH, hepatocellular ballooning, and necroinflammation. Importantly, the association between the E167K variant and advanced fibrosis was abolished after conditioning for NASH, suggesting that fibrosis progression is mediated by the effect of the genetic variant on intracellular retention of lipids, mainly triglycerides and cholesterol, within hepatocytes. In keeping with this interpretation, the severity of liver damage was found to be correlated with the amount of hepatic triglycerides accumulation in patients with NAFLD [89].

The link between the* NCAN* locus and NAFLD severity [90, 91] and an independent study reporting an association between* TM6SF2* and moderate/severe fibrosis [92] support the association between* TM6SF2* and liver damage in NAFLD. Indeed, the E167K is the causal variant explaining the association of the* NCAN* locus with altered lipid metabolism [11, 87].

The association of E167K with NASH and advanced fibrosis contradicts the notion that long-term storage of fatty acids in hepatocytes in triglycerides is benign [93]. In addition, it has been demonstrated that also the* PNPLA3* I148M variant by altering lipid droplets remodelling impairs the ability to export triglycerides to secreted VLDL [45, 46]. The deleterious effect on liver damage of the impaired ability to secrete triglycerides in VLDL is also supported by the association between progressive liver disease and rare* apolipoprotein B (APOB)* and* microsomal triglyceride transfer protein (MTTP)* mutations directly causing VLDL retention [93, 94]. A possible alternative mechanism may be related to the toxicity of excessive hepatocellular cholesterol and the consequent mitochondrial damage in carriers of the E167K variant [95, 96]. All in all, these novel findings suggest that compartmentalization of neutral lipids within hepatocytes is harmful for the liver.

## 6. *GCKR *and Lipogenesis

In a meta-analysis of combined GWAS datasets, besides* PNPLA3* I148M, other genetic loci were associated with liver fat content. These included* NCAN* (explained by* TM6SF2*, as mentioned previously),* glucokinase regulator* (*GCKR*, SNP rs780094), and* lysophospholipase-like 1* (*LYPLAL1*, SNP rs12137855). Both variants in* GCKR*, a regulator of glucose metabolism and* LYPLAL1*, involved in triglycerides catabolism, were also shown to influence liver damage [97, 98]. It has been hypothesized that the association of the rs780094* GCKR* polymorphism with hepatic fat accumulation can be explained by the linkage disequilibrium with rs1260326, encoding for the P446L protein variant. The P446L variant indeed affects GCKR ability to negatively regulate glucokinase in response to fructose-6-phosphate, thereby determining constitutive activation of hepatic glucose uptake [99]. This would lead to decreased circulating fasting glucose and insulin levels, but on the other hand it would lead to increased glycolysis and production of malonyl-CoA. Malonyl-CoA is a key metabolite, because it favors hepatic fat accumulation by serving as a substrate for lipogenesis and blocks fatty acid oxidation through the inhibition of carnitine-palmitoyl transferase-1. The combined effects of* PNPLA3* I148M and* GCKR* P446L polymorphisms explained up to one-third of variability in liver fat content in a recently reported series of obese children [100, 101]. All this body of evidence shows that genetic variants influencing hepatocellular lipid accumulation predispose both to fatty liver and to progressive NASH.

## 7. Other Variants Regulating Lipid Metabolism

According to the aforementioned hypothesis, other genes involved in hepatic fat uptake, synthesis, storage, and mobilization are therefore candidates to influence the development and progression of NAFLD. Among these are variants in peroxisome proliferator-activated nuclear receptors (PPAR). PPAR*α*, a molecular target of long chain fatty acids, eicosanoids, and fibrates [102], is highly expressed in tissues that catabolize fatty acids such as the liver, where, under condition of increased hepatic fatty acid influx or decreased fatty acid efflux, PPAR*α* activation prevents the accumulation of triglycerides by increasing the rate of fatty acid catabolism. However, the Leu162Val loss-of-function PPAR*α* variant did not influence the risk of NAFLD and the severity of liver disease [103, 104].

PPAR*γ* is highly expressed in adipose tissue and regulates adipocyte differentiation, FFA uptake, and storage. Pharmacological activation of PPAR*γ* improves insulin resistance in diabetes and decreases steatosis in NAFLD patients by restoring adipose tissue insulin sensitivity and adiponectin release [105, 106]. The Pro12Ala loss-of-function SNP in* PPARγ2*, inducing a modest impairment of transcriptional activity due to decreased DNA-binding affinity, was associated with a reduction of PPAR*γ* activity in adipose tissue as well as decreased insulin resistance [107]. However, inconsistent results have been reported concerning the association of this variant with the severity of liver damage in NAFLD [103, 104].

Another interesting candidate is represented by* Lipin1* (*LPIN1*), a phosphatidate phosphatase that is highly expressed in the liver and adipose tissue, and is involved in the synthesis of phospholipids downstream of the step catalysed by PNPLA3 [108] and of triglycerides. LPIN1 is required for adipogenesis and the normal metabolic flux between adipose tissue and liver, where it also acts as an inducible transcriptional coactivator to regulate fatty acid metabolism [56].* LPIN1* variants have been associated with several components of the metabolic syndrome, including body mass, insulin levels, resting metabolic rate, and responsiveness to insulin sensitizers [109, 110]. In a pediatric population,* LPIN1* rs13412852 TT genotype was protective towards NAFLD [111]. Although independent validation of these results is required, these data suggest that* LPIN1* variants predispose to progressive NASH at early age by influencing lipogenesis and lipid metabolism.

Fatty acid transport proteins (FATP) hold a crucial role in mediating cellular FFA uptake. In the liver FATP2 and FATP5 are predominantly expressed [112].* FATP5* encodes a multifunctional protein which increases the hepatic FFA uptake and activates very long-chain fatty acids and has bile-CoA ligase activity [113, 114].* FATP5* silencing reversed diet-induced NAFLD and improved hyperglycemia in mice [115]. Interestingly, the rs56225452* FATP5* promoter variant linked to transcriptional activity has been associated with ALT levels in a population study and with steatosis severity in NAFLD patients [116].

Interestingly, a genetic variant in the promoter region regulating the expression of uncoupling protein 2 (UCP2) has recently been associated with altered lipoprotein metabolism and reduced susceptibility to NASH [117]. Besides regulating mitochondrial redox status and energy dissipation, UCP2 is involved in the export of fatty acids from mitochondria in hepatocytes and is induced during steatosis. It is therefore possible that it may modulate the risk of NASH by altering mitochondrial fluxes of lipids during NAFLD.

Apolipoprotein C3 (APOC3) is a major constituent of VLDL, chylomicrons, and HDL cholesterol, which inhibits lipoprotein lipase and triglyceride clearance [118]. Petersen et al. reported that two common* APOC3* T-455C and C-482T promoter variants predispose to liver fat accumulation in Indian individuals. However, these data have not been confirmed in large population studies and in other ethnic groups. Furthermore, APOC3 variants were not associated with the histological severity of liver damage in NAFLD [119–121]. These data suggest that genetic factors influencing triglyceride metabolism outside the liver are not involved in the pathogenesis of progressive NAFLD.

## 8. Other Variants Not Implicated in Lipid Metabolism

Although recent genetic evidence concordantly points to a major role of variants influencing intrahepatocellular lipid metabolism in the pathogenesis of NAFLD and progressive NASH, as recently reviewed by our group [20], other genetic variants may be implicated in the progression of liver disease.

The most solid associations have been collected for genetic variants involved in the regulation of inflammation, such as* TNFalpha* and* IL28B* polymorphisms [13, 122], oxidative stress [13, 122], iron metabolism [15, 17, 18], which is frequently altered in NAFLD [124], and fibrogenesis [19]. However, the overall evidence supporting these associations is weaker than that already obtained for the* PNPLA3* I148M and* TM6SF2* E167K genetic variants, as these results still need independent validation in independent cohorts.

## 9. Conclusions

Genes play a key role in the susceptibility and progression of NAFLD. To date, the* PNPLA3 *I148M and* TM6SF2* E167K gene variants are the major determinants of interindividual differences in liver steatosis and susceptibility to progressive NASH. Both of these genes determine liver fat retention through lipid droplets and very low-density lipoproteins modifications. PNPLA3 affects also directly hepatic stellate cells and retinol metabolism. These novel findings suggest that hepatocellular accumulation of neutral lipids is harmful for the liver. Several other genetic variants, including rare mutations, involved in the regulation of hepatocellular lipid metabolism, are being scrutinized.

Future challenges will be (a) to understand the molecular mechanisms underlying the association between gene variants and progressive liver disease, (b) to evaluate the impact of gene variants in the clinical practice to stratify individual risk, and (c) to examine pharmacogenetic response to available therapies. This knowledge will offer insights into pathogenesis of NASH and importantly suggest novel therapeutic targets.
